# Prognostic impact of bridge or neoadjuvant induction chemotherapy in patients with resected oral cavity cancer: A nationwide cohort study

**DOI:** 10.1002/cam4.70061

**Published:** 2024-08-05

**Authors:** Cheng‐Lung Hsu, Yu‐Wen Wen, Hung‐Ming Wang, Chia‐Hsun Hsieh, Chi‐Ting Liao, Li‐Yu Lee, Shu‐Hang Ng, Chien‐Yu Lin, Wen‐Cheng Chen, Jin‐Ching Lin, Yao‐Te Tsai, Shu‐Ru Lee, Chih‐Yen Chien, Chun‐Hung Hua, Cheng Ping Wang, Tsung‐Ming Chen, Shyuang‐Der Terng, Chi‐Ying Tsai, Kang‐Hsing Fan, Chih‐Hua Yeh, Chih‐Hung Lin, Chung‐Kan Tsao, Nai‐Ming Cheng, Tuan‐Jen Fang, Shiang‐Fu Huang, Chung‐Jan Kang, Li‐Ang Lee, Ku‐Hao Fang, Yu‐Chien Wang, Wan‐Ni Lin, Li‐Jen Hsin, Tzu‐Chen Yen, Chun‐Ta Liao

**Affiliations:** ^1^ Department of Medical Oncology Chang Gung Memorial Hospital and Chang Gung University Taoyuan Taiwan; ^2^ Clinical Informatics and Medical Statistics Research Center Chang Gung University Taoyuan Taiwan; ^3^ Division of Thoracic Surgery Chang Gung Memorial Hospital Taoyuan Taiwan; ^4^ Department of Pathology Chang Gung Memorial Hospital and Chang Gung University Taoyuan Taiwan; ^5^ Department of Diagnostic Radiology Chang Gung Memorial Hospital and Chang Gung University Taoyuan Taiwan; ^6^ Department of Radiation Oncology Chang Gung Memorial Hospital and Chang Gung University Taoyuan Taiwan; ^7^ Department of Radiation Oncology Chang Gung Memorial Hospital Chiayi Taiwan; ^8^ Department of Radiation Oncology Changhua Christian Hospital Changhua Taiwan; ^9^ Department of Otorhinolaryngology‐Head and Neck Surgery Chang Gung Memorial Hospital Chiayi Taiwan; ^10^ Research Service Center for Health Information Chang Gung University Taoyuan Taiwan; ^11^ Department of Otolaryngology, Chang Gung Memorial Hospital Kaohsiung Medical Center Chang Gung University College of Medicine Kaohsiung Taiwan; ^12^ Department of Otorhinolaryngology China Medical University Hospital Taichung Taiwan; ^13^ Department of Otolaryngology National Taiwan University Hospital and College of Medicine Taipei Taiwan; ^14^ Department of Otolaryngology Shuang Ho Hospital, Taipei Medical University New Taipei City Taiwan; ^15^ Department of Head and Neck Surgery Koo Foundation Sun Yat‐Sen Cancer Center Taipei Taiwan; ^16^ Department of Oral and Maxillofacial Surgery Chang Gung Memorial Hospital, Chang Gung University Taoyuan Taiwan; ^17^ Department of Radiation Oncology New Taipei Municipal TuCheng Hospital New Taipei City Taiwan; ^18^ Department of Plastic and Reconstructive Surgery Chang Gung Memorial Hospital and Chang Gung University Taoyuan Taiwan; ^19^ Department of Nuclear Medicine and Molecular Imaging Center Chang Gung Memorial Hospital and Chang Gung University Taoyuan Taiwan; ^20^ Department of Otorhinolaryngology, Head and Neck Surgery Chang Gung Memorial Hospital and Chang Gung University Taoyuan Taiwan

**Keywords:** cancer registry, clinical outcomes, induction chemotherapy, oral cavity squamous cell carcinoma

## Abstract

**Background:**

While surgery remains the primary treatment for oral squamous cell carcinoma (OCSCC), induction chemotherapy (IC) can be used as a bridging or neoadjuvant therapy. This nationwide study in Taiwan examines the survival outcomes of OCSCC patients who received IC before surgery.

**Methods:**

We analyzed data from 29,891 patients with OCSCC. Of these, 29,058 initially underwent surgery (OP group), whereas 833 received IC before surgery (IC + OP group). A propensity score (PS)‐matched analysis (4, 1 ratio, 3260 vs. 815 patients) was performed considering tumor subsite, sex, age, Charlson comorbidity index, clinical T1–T4b tumors, clinical N0–3 disease, and clinical stage I–IV.

**Results:**

In the PS‐matched cohort, the 5‐year disease‐specific survival (DSS) and overall survival (OS) rates were 65% and 57%, respectively. When comparing the OP and IC + OP groups, the 5‐year DSS rates were 66% and 62%, respectively (*p* = 0.1162). Additionally, the 5‐year OS rates were 57% and 56%, respectively (*p* = 0.9917). No significant intergroup differences in survival were observed for specific subgroups with cT4a tumors, cT4b tumors, cN3 disease, pT4b tumors, and pN3 disease. However, for patients with pT4a tumors, the OP group demonstrated superior 5‐year outcomes compared to the IC + OP group, with a DSS of 62% versus 52% (*p* = 0.0006) and an OS of 53% versus 44% (*p* = 0.0060). Notably, patients with cT2–3, cN1, and c‐Stage II disease in the IC + OP group were significantly more likely to achieve pT0–1 status (*p* < 0.05).

**Conclusions:**

Following PS matching, the IC + OP group generally exhibited similar prognosis to the OP group. However, for pT4a tumors, the OP group showed superior 5‐year outcomes. While IC may not universally improve survival, it could be advantageous for patients who respond positively to the treatment.

## 1 INTRODUCTION

The National Comprehensive Cancer Network (NCCN) guidelines recommend initial surgery, which may include neck dissection, as the primary treatment for oral cavity squamous cell carcinoma (OCSCC).^1^ Depending on the presence of pathological risk factors, adjuvant chemoradiotherapy (CRT) or radiotherapy (RT) may follow the operation.^1^, ^2^ In cases where tumors are initially unresectable, neoadjuvant induction chemotherapy (IC) can be administered. If the tumor responds favorably, surgery may then be considered.^3^, ^4^, ^5^, ^6^ For patients experiencing delays in their surgical schedule, IC can also serve as a bridging treatment to prevent tumor growth during the waiting period. Furthermore, some patients and head and neck surgeons may opt for neoadjuvant IC to shrink the tumor size. This approach can lead to less invasive surgery, thereby helping to preserve oral function.^7^, ^8^, ^9^ However, it is important to note that the primary objective of neoadjuvant or bridging chemotherapy in OCSCC is to target the primary tumor rather than cervical lymph node involvement. Consequently, radical surgery and neck dissection are frequently required, particularly in advanced cases.^10^, ^11^ After chemotherapy, a minority of patients may achieve either a complete or partial response^3^, ^4^, ^8^, ^9^, ^12^ or even a pathological complete response (pCR).^7^, ^11^ Expectedly, this group tends to have better survival outcomes compared to other patients.

A randomized trial involving 195 patients with resectable OCSCC compared upfront resection to 3 cycles of IC using cisplatin and 5‐fluorouracil, followed by resection. Both groups received appropriate adjuvant therapy. Although no overall survival (OS) benefit was observed in the IC group, the need for mandibulectomy and/or postoperative RT was significantly reduced compared to the control arm.^10^ Instead of focusing on improving survival endpoints, pursuing less morbid surgical interventions or reducing the need for or intensity of adjuvant therapy could be potential justifications for using IC in OCSCC.

To our knowledge, no large studies have been published that directly compared the outcomes between patients with OCSCC who had undergone primary surgery (OP group) with those who had received bridging or neoadjuvant IC followed by surgery (IC + OP group). To address this gap, we conducted a nationwide study in Taiwan with three primary objectives. First, we sought to ascertain whether a survival difference exists between patients receiving primary surgery alone and those undergoing IC followed by surgery. Second, in the event that both groups demonstrate similar prognoses, we aimed to identify specific subgroups that exhibit divergent outcomes between these two treatment strategies. Finally, we endeavored to pinpoint patients who are more likely to respond favorably to IC, as this could potentially lead to reduced surgical morbidity and/or improved survival outcomes.

### 1.1 Data sources

We sourced patient data from the “long‐form” of the Taiwanese Cancer Registry Database (TCRD), which covers over 99% of Taiwanese patients diagnosed with OCSCC. However, the TCRD lacks data on specific chemotherapy protocols, tumor resectability, postoperative morbidity and its severity, residual disease following surgery, and salvage treatment for patients who experience disease recurrence. Survival outcome data were gathered from the Taiwanese National Health Insurance Research Dataset (TNHIRD). The study adheres to the Reporting Recommendations for Tumor Marker Prognostic Studies (REMARK) guidelines.^13^, ^14^ The research protocol was approved by the Ethics Committee of Chang Gung Memorial Hospital (reference number: 201801398B0A3), which also granted a waiver for obtaining written informed consent.

### 1.2 Patient selection

**FIGURE 1:**
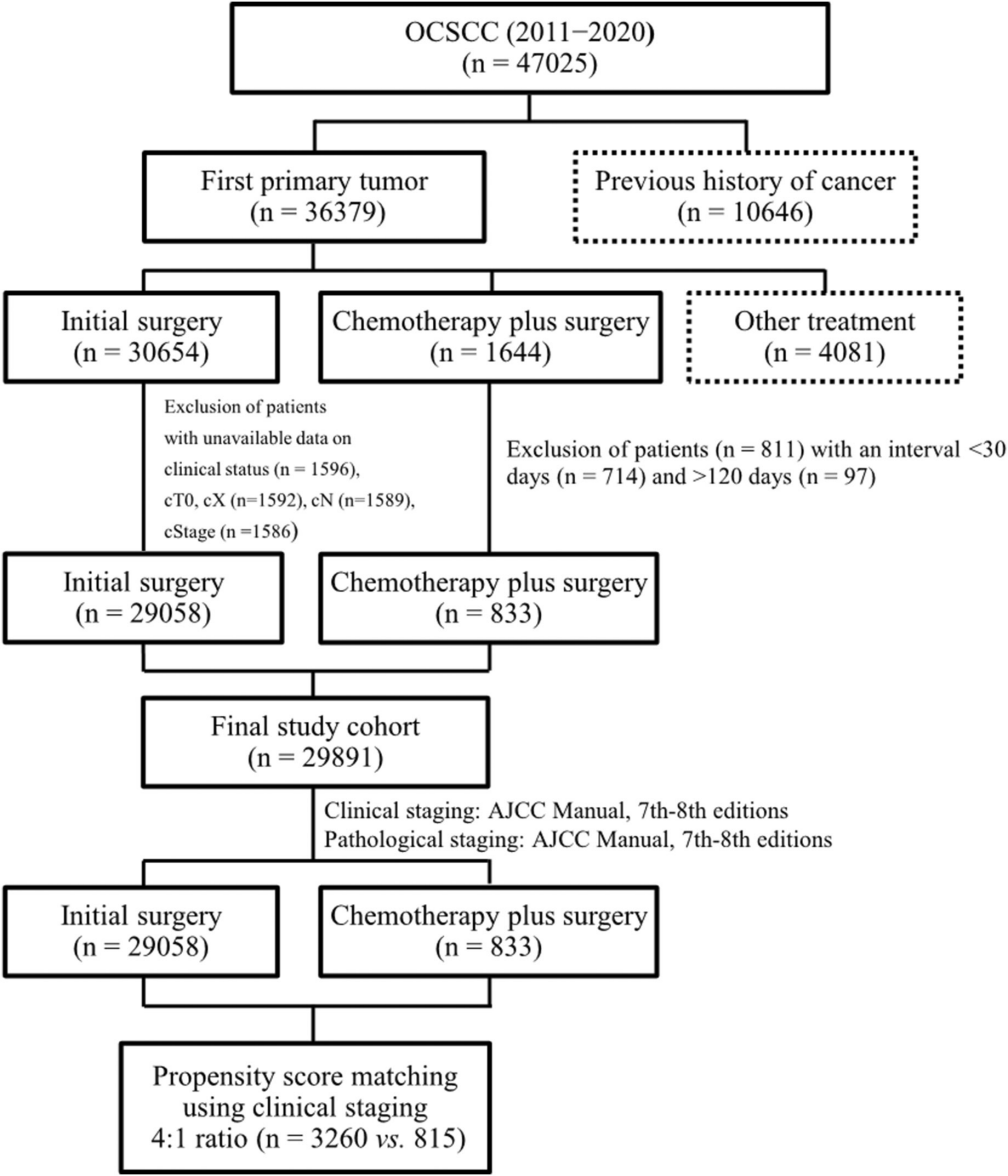
Patient progression through the study.

This study considered patients diagnosed with OCSCC between 2011 and 2020 (*n* = 47,025) for inclusion. Selection was guided by the International Classification of Diseases for Oncology, Third Edition (ICD‐O‐3) codes. The study flow chart (Figure 1) provides details about the inclusion and exclusion of cases. Patients were deemed ineligible if their medical records indicated any of the following: (1) prior history of cancer, (2) initial nonsurgical treatment, excluding cases where chemotherapy was followed by surgery, (3) unknown clinical stage, or (4) interval between IC and surgery of less than 30 days or more than 120 days. The final study cohort consisted of 29,891 patients, all of Taiwanese descent. As patient selection spanned from 2011 and 2020, tumor staging was performed according to the criteria outlined in the AJCC Staging Manual, Seventh Edition (2010) and Eighth Edition (2018). The Eighth Edition incorporates depth of invasion (DOI) and extranodal extension (ENE) into the staging process.^15^ Out of 29,891 OCSCC patients, 29,058 (97%) underwent initial surgery, while 833 (3%) received bridging or neoadjuvant IC. Subsequently, a propensity score (PS)‐matched analysis (4, 1 ratio, 3260 vs. 815 patients) was implemented considering tumor subsite, sex, age, Charlson comorbidity index (CCI), clinical T1–T4b tumors, clinical N0–3 disease, and clinical stage I–IV. Pathological parameters—including margin status, tumor differentiation, pT status, pN status, and pStage—were not included in the PS matching process. Notably, DOI information was missing for 12% (3501/29058) of patients in the OP group and 77% (643/833) of patients in the IC + OP group. Given the substantial limitation posed by the incomplete DOI data, particularly in the IC + OP group, we deliberately excluded DOI as an analysis parameter in this study. The follow‐up period was calculated from the day of surgery to either the patient's death or the conclusion of the study (December 2021).

### 1.3 Data collection

We acquired study variables from the 2020 TCRD release and the 2021 TNHIRD release, and conducted final data analyses in May 2024. We obtained morbidity and mortality data related to OCSCC from the TNHIRD and used the extracted information to calculate disease‐specific survival (DSS) and OS, respectively. The TCRD generally adheres to the guidelines delineated in the Standards for Oncology Registry Entry (STORE) manual.^16^ As per the STORE guidelines, data on disease recurrences were collected independently from the anatomical site (i.e., local, regional, or distant recurrences); furthermore, only the initial recurrence was documented. Each participating hospital transmitted the relevant information to the TCRD during the first and fifth years of patient follow‐up. Consequently, the survival data were considered entirely reliable for the calculation of DSS and OS; however, this reliability did not extend to disease‐free survival (DFS), which includes the analysis of local control, neck control, and distant metastases.

### 1.4 Statistical analysis

We utilized the Kaplan–Meier method to generate survival curves, with the log‐rank test applied for statistical comparisons. We applied univariable and multivariable Cox proportional hazards regression analyses to investigate the relationships between the study variables and survival outcomes. Using a stepwise selection method, we incorporated all parameters from the univariable analysis into the multivariable model. The findings are presented as hazard ratios (HRs) accompanied by their corresponding 95% confidence intervals (CIs). We considered two‐tailed *p* values as significant when they were less than 0.05.

## 2 RESULTS

### 2.1 Patient characteristics

Table 1 presents the general characteristics of patients in the OP and IC + OP groups. Before PS matching, the IC + OP group showed a significantly higher prevalence of several variables compared to the OP group, including: (1) tumors subsites other than the tongue and buccal mucosa, (2) male sex, (3) cT4a and cT4b tumors, (4) cN2 and cN3 disease, and (5) c‐Stage IV. Notably, the IC + OP group had a lower CCI compared to the OP group. After PS matching for tumor subsite, sex, age, CCI, clinical T‐status, clinical N disease, and clinical stage, the IC + OP group showed a significantly higher prevalence of several pathological variables compared to the OP group. These parameters included well‐differentiated malignancies, pT0 and pT1 tumors, and p‐Stage I disease. Interestingly, the IC + OP group had fewer patients receiving free flap reconstruction and CRT.

**TABLE 1:** General characteristics of patients with oral cavity squamous cell carcinoma who had undergone initial surgery versus bridge/neoadjuvant induction chemotherapy followed by surgery.

Characteristic (*n*, %)	Original cohort				Propensity score‐matched cohort			
	Initial surgery (*n* = 29,058)	Induction chemotherapy plus surgery (*n* = 833)	SMD (%)	*p*	Initial surgery (*n* = 3260)	Induction chemotherapy plus surgery (*n* = 815)	SMD (%)	*p*
Tumor subsite								
Tongue (11,083, 37.1)	10,865 (37.4)	218 (26.2)	24.27	<0.0001	919 (28.2)	215 (26.4)	4.06	0.1460
Buccal (10,388, 34.8)	10,071 (34.7)	317 (38.1)	−7.07		1163 (35.7)	308 (37.8)	−4.39	
Other sites (8420, 28.1)	8122 (27.9)	298 (35.7)	−16.85		1178 (36.1)	292 (35.8)	0.64	
Sex								
Men (26,896, 90.0)	26,108 (89.8)	788 (94.6)	−17.81	<0.0001	3104 (95.2)	770 (94.5)	3.33	0.3336
Women (2995, 10.0)	2950 (10.2)	45 (5.4)	17.81		156 (4.8)	45 (5.5)	−3.33	
Age, years (mean ± SD)	55.63 ± 11.34	53.36 ± 10.04	21.20	<0.0001	53.12 ± 9.86	53.60 ± 9.96	−4.86	0.0031
CCI (mean ± SD)	0.80 ± 1.17	0.57 ± 0.91	21.94	<0.0001	0.53 ± 0.86	0.57 ± 0.91	−4.52	0.0016
Clinical T status								
T1 (9312, 31.2)	9293 (32.0)	19 (2.3)	85.77	<0.0001	78 (2.4)	19 (2.3)	0.40	0.0759
T2 (10,020, 33.5)	9906 (34.1)	114 (13.7)	49.29		461 (14.1)	114 (14.0)	0.44	
T3 (2553, 8.5)	2470 (8.5)	83 (10.0)	−5.06		340 (10.4)	83 (10.2)	0.81	
T4a (6844, 22.9)	6424 (22.1)	420 (50.4)	−61.62		1744 (53.5)	420 (51.5)	3.93	
T4b (1162, 3.9)	965 (3.3)	197 (23.6)	−62.34		637 (19.6)	179 (22.0)	−5.98	
Clinical N status								
N0 (19,145, 64)	18,940 (65.2)	205 (24.6)	89.33	<0.0001	807 (24.8)	205 (25.2)	−0.92	0.2292
N1 (4232, 14.2)	4100 (14.1)	132 (15.8)	−4.87		480 (14.7)	132 (16.2)	−4.07	
N2 (6182, 20.7)	5735 (19.7)	447 (53.7)	−75.2		1825 (56.0)	441 (54.1)	4.50	
N3 (332, 1.1)	283 (1.0)	49 (5.9)	−27.23		148 (4.5)	37 (4.5)	1.19	
Clinical stage								
I (8293, 27.7)	8280 (28.5)	13 (1.6)	81.37	<0.0001	58 (1.8)	13 (1.6)	1.43	0.2352
II (6776, 22.7)	6738 (23.2)	38 (4.6)	55.95		148 (4.5)	38 (4.7)	−0.59	
III (4014, 13.4)	3953 (13.6)	61 (7.3)	20.63		254 (7.8)	61 (7.5)	1.15	
IV (10,808, 36.2)	10,087 (34.7)	721 (86.5)	−125.18		2800 (85.9)	703 (86.2)	−1.06	
Margin status, mm								
Positive (1857, 6.2)	1775 (6.1)	82 (9.8)	−13.82	<0.0001	313 (10.2)	80 (11.4)	−3.77	0.2390
<5 (13,104, 43.8)	12,775 (44.0)	329 (39.5)	9.07		1344 (43.8)	325 (46.2)	−4.80	
≥5 (11,906, 39.8)	11,599 (39.9)	307 (36.9)	6.3		1413 (46.0)	299 (42.5)	7.16	
Unknown (3024, 10.2)	2909 (10.0)	115 (13.8)	−11.74					
Differentiation								
Well differentiated (8797, 29.4)	8603 (29.6)	194 (23.3)	14.36	<0.0001	784 (24.6)	190 (29.9)	−12.03	0.0020
Moderately differentiated (17,610, 58.9)	17,202 (59.2)	408 (49.0)	20.62		2077 (65.1)	399 (62.8)	4.74	
Poorly differentiated (2284, 7.7)	2237 (7.7)	47 (5.6)	8.25		329 (10.3)	46 (7.2)	10.86	
Unknown (1200, 4.0)	1016 (3.5)	184 (22.1)	−57.95					
Pathologic T status				<0.0001	3239	739		<0.0001
T0 (321, 1.0)^a^	242 (0.9)	79 (9.4)	39.88		21 (0.5)	76 (9.4)	−40.71	
T1 (10,731, 35.9)	10,583 (36.4)	148 (17.8)	42.93		211 (6.5)	146 (17.9)	−35.51	
T2 (8913, 29.8)	8769 (30.2)	144 (17.3)	30.65		620 (19.0)	143 (17.5)	3.81	
T3 (2958, 9.9)	2887 (9.9)	71 (8.5)	4.88		468 (14.4)	71 (8.7)	17.74	
T4a (6208, 20.8)	5886 (20.3)	322 (38.7)	−41.21		1560 (47.9)	314 (38.5)	18.91	
T4b (760, 2.6)	691 (2.4)	69 (8.3)	−26.52		380 (11.7)	65 (8.0)	12.40	
Pathologic N status								
pNx (5735, 19.2)	5708 (19.6)	27 (3.2)	53.33	<0.0001	110 (3.4)	26 (3.2)	1.03	0.4083
pN0 (16,215, 54.2)	15,785 (54.3)	430 (51.6)	5.42		1638 (50.2)	425 (52.1)	−3.81	
pN1 (2752, 9.2)	2665 (9.2)	87 (10.4)	−4.28		320 (9.8)	84 (10.3)	−1.63	
pN2 (4233, 14.2)	4020 (13.8)	213 (25.6)	−29.83		935 (28.7)	208 (25.5)	7.11	
pN3 (956, 3.2)	880 (3.1)	76 (9.2)	−25.72		257 (7.9)	72 (8.9)	−3.44	
Pathologic stage								
I (9368, 31.3)	9281 (31.9)	87 (10.4)	54.52	<0.0001	167 (5.2)	87 (11.6)	−23.42	<0.0001
II (6196, 20.7)	6120 (21.1)	76 (9.1)	33.82		349 (10.8)	75 (10.0)	2.59	
III (3744, 12.5)	3660 (12.6)	84 (10.1)	7.93		370 (11.5)	84 (11.2)	0.75	
IV (9842, 32.9)	9326 (32.1)	516 (61.9)	−62.67		2340 (72.5)	502 (67.1)	11.84	
Unknown (741, 2.6)	671 (2.3)	70 (8.5)	−27.32					
Use of a free flap								
No (14,899, 49.8)	14,681 (50.5)	218 (26.2)	51.73	<0.0001	732 (22.5)	213 (26.1)	−8.59	0.0182
Yes (14,992, 50.2)	14,377 (49.5)	615 (73.8)	−51.73		2528 (77.5)	602 (73.9)	8.59	
Adjuvant therapy after surgery								
None (16,577, 55.5)	16,407 (56.5)	170 (20.4)	79.8	<0.0001	813 (24.9)	169 (20.7)	10.02	<0.0001
Chemotherapy (991, 3.3)	945 (3.3)	46 (5.5)	−11.1		107 (3.3)	46 (5.6)	−11.46	
Radiotherapy (4102, 13.7)	3854 (13.3)	248 (29.8)	−41.01		548 (16.8)	241 (29.6)	−30.59	
Chemotherapy plus radiotherapy (8221, 27.5)	7852 (26.9)	369 (44.3)	−36.67		1792 (55.0)	359 (44.0)	21.97	

Abbreviations: CCI, Charlson comorbidity index; SD, standard deviation; SMD, standardized mean difference.

^a^ pT0: the initial biopsy results confirmed the presence of squamous cell carcinoma; however, subsequent surgery revealed no residual tumor tissue.

### 2.2 Five‐year survival rates

**FIGURE 2:**
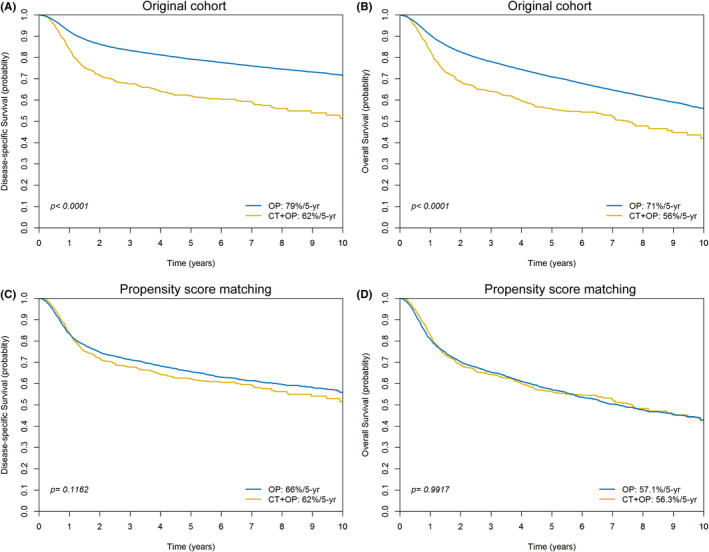
Kaplan–Meier plots comparing disease‐specific survival before (A, B) and after (C, D) propensity score matching in patients who initially underwent surgery (OP group) and those who received induction chemotherapy prior to surgery (IC + OP group).

In the initial cohort of 29,891 patients, the 5‐year survival outcomes were compared between the OP group and the IC + OP group. The DSS rate was 79% for the OP group and 62% for the IC + OP group (*p* < 0.0001, Figure 2A). In addition, the OS rate was 71% for the OP group and 56% for the IC + OP group (*p* < 0.0001, Figure 2B). In the propensity score‐matched cohort of 4075 patients, the 5‐year survival outcomes were again compared between the OP group and the IC + OP group. The DSS rate was 66% for the OP group and 62% for the IC + OP group (*p* = 0.1162, Figure 2C). Furthermore, the OS rate was 57% for the OP group and 56% for the IC + OP group (*p* = 0.9917).

### 2.3 Five‐year survival rates for patients with advanced disease in the propensity score‐matched cohort

**FIGURE 3:**
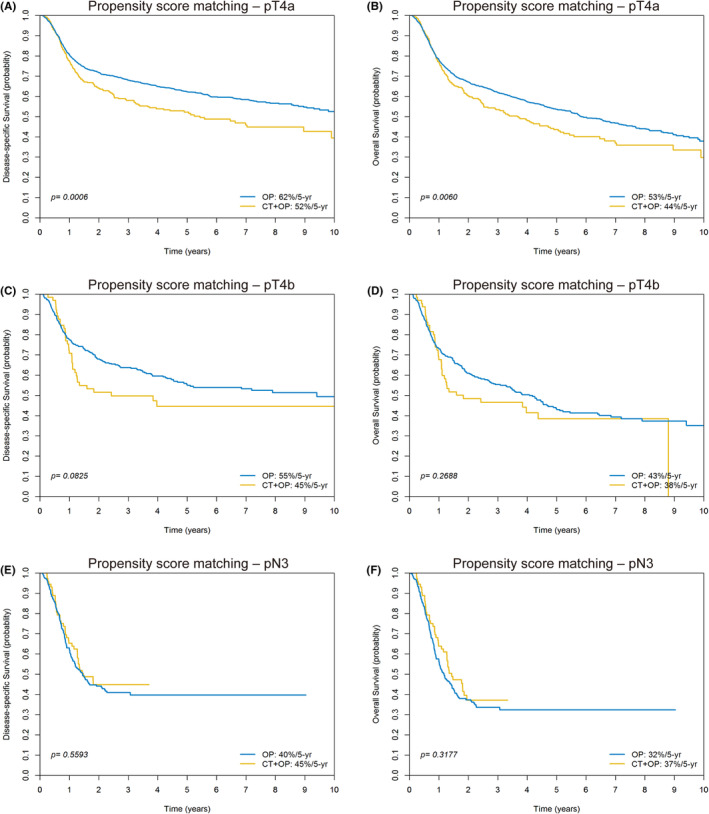
Kaplan–Meier plots of disease‐specific survival and overall survival for patients with pT4a (A, B), pT4b (C, D), and pN3 (E, F) tumors in the propensity score‐matched cohort.

After PS matching, the 5‐year outcomes of the OP and IC + OP groups were compared in the context of advanced disease. The results observed for specific subgroups were as follows: for cT4a tumors, DSS, 63% versus 59%, *p* = 0.1560 (Figure S1A); OS, 54% versus 53%, *p* = 0.9163 (Figure S1B); for cT4b tumors, DSS, 60% versus 63%, *p* = 0.3109 (Figure S1C); OS, 51% versus 58%, *p* = 0.1011 (Figure S1D); for cN3 tumors, DSS, 48% versus 36%, *p* = 0.8374 (Figure S1E); OS, 38% versus 30%, *p* = 0.6679 (Figure S1F); for pT4a tumors, DSS, 62% versus 52%, *p* = 0.0006 (Figure 3A); OS, 53% versus 44%, *p* = 0.0060 (Figure 3B); for pT4b tumors, DSS, 55% versus 45%, *p* = 0.0825 (Figure 3C); OS, 43% versus 38%, *p* = 0.2688 (Figure 3D); for pN3 tumors, DSS, 40% versus 45%, *p* = 0.5593 (Figure 3E); OS, 32% versus 37%, *p* = 0.3177 (Figure 3F). No significant intergroup differences in survival were observed for specific subgroups, except pT4a tumors.

### 2.4 Factors influencing five‐year survival outcomes for patients with pT4a tumors in the propensity score‐matched cohort

Following PS matching, the pT4 tumor category comprised 1560 patients in the OP group and 314 patients in the IC + OP group. An analysis of the factors influencing the 5‐year survival outcomes for patients with pT4a tumors is presented in Table 2. For DSS, statistically significant differences between groups were observed for the following variables: buccal subsite, male sex, margin status less than 5 mm, well‐to‐moderate tumor differentiation, and pN0 disease. In terms of OS, significant differences were noted for buccal subsite, male sex, positive margins, margin status less than 5 mm, well‐differentiated tumors, and pN0 disease.

**TABLE 2:** General characteristics of patients with pT4a oral cavity squamous cell carcinoma who had undergone initial surgery versus bridge/neoadjuvant induction chemotherapy followed by surgery (*n* = 1874): Associations with 5‐year clinical outcomes.

Characteristic (*n*, %)	Number of patients		5‐year disease‐specific survival			5‐year overall survival		
	Initial surgery (*n*)	Induction chemotherapy plus surgery (*n*)	Initial surgery (%)	Induction chemotherapy plus surgery (%)	*p*	Initial surgery (%)	Induction chemotherapy plus surgery (%)	*p*
Tumor subsite								
Tongue (462, 24.7)	395	67	51	41	0.1723	42	32	0.2140
Buccal (583, 31.1)	471	112	69	56	0.0045	61	50	0.0136
Gum (52.5, 28.0)	436	89	67	58	0.0715	57	44	0.0815
Other sites (304, 16.2)	258	46	58	48	0.2217	50	46	0.7800
Sex								
Men (1789, 95.5)	1494	295	62	52	0.0007	53	43	0.0083
Women (85, 4.5)	66	19	69	57	0.4941	62	45	0.3832
Margin status (mm)								
Positive (208, 11.1)	167	41	46	29	0.1012	37	11	0.0147
<5 (778, 41.5)	633	145	61	50	0.0016	53	44	0.0205
≥5 (802, 42.8)	690	112	67	64	0.6803	58	54	0.8948
Unknown (86, 4.6)								
Differentiation								
Well differentiated (440, 23.5)	358	82	76	60	0.0032	68	47	0.0033
Moderately differentiated (1183, 63.1)	1023	160	60	55	0.0281	52	46	0.0525
Poorly differentiated (174, 9.3)	158	16	42	36	0.4657	33	36	0.9105
Unknown (77, 4.1)								
Pathologic N status								
pNx (10, 0.5)	9	1	67	0	0.0694	56	0	0.2240
pN0 (936, 49.9)	785	151	79	66	<0.0001	71	58	0.0013
pN1 (163, 8.7)	135	28	58	56	0.7542	50	45	0.9945
pN2 (584, 31.2)	486	98	46	37	0.1548	35	30	0.3611
pN3 (181, 9.7)	145	36	33	38	0.4195	26.5	26.7	0.4024

### 2.5 Five‐year survival rates for patients who achieved pathological complete response in the propensity score‐matched cohort

In the PS‐matched cohort (Table 1), 45 patients successfully achieved a pCR (pT0N0). The 5‐year DSS rate was 95% for patients who achieved pCR, compared to 60% for those who did not (*n* = 770; *p* = 0.0001). Similarly, the OS rate was 95% for patients who achieved pCR, compared to 54% for those who did not (*p* < 0.0001).

### 2.6 Factors influencing pathological complete response (pT0N0) or pT0–1 status in the induction chemotherapy arm following propensity score matching

To identify subgroups that might benefit from IC, we conducted a comprehensive analysis of patients who achieved pCR (pT0N0) or pT0–1 status in the IC arm following PS matching (*n* = 815). The main findings are summarized in Table 3. Notably, 45 patients achieved pCR (pT0N0), whereas 770 did not. In addition, 222 patients achieved pT0–1 tumor status, while 593 did not. We identified several patient subgroups that derived significant benefit from IC (*p* < 0.05) in terms of achieving pT0–1 status, including those with cT2–3, cN1, and c‐Stage II disease. Furthermore, certain subgroups demonstrated a trend towards benefiting from IC (*p* > 0.05), such as patients with tumors in the buccal subsite and female patients, when considering pT0–1 status. Notably, when evaluating pCR status, subgroups that might benefit from IC included those with tumors in the tongue and buccal subsites, female patients, and those with cT2–3, cN1, and c‐Stage II disease. As anticipated, the achievement of pCR or pT0–1 status in the OP + IC group was associated with a reduced need for free flap reconstruction and adjuvant RT/CRT.

**TABLE 3:** General characteristics of propensity score‐matched patients with oral cavity squamous cell carcinoma who had undergone bridge/neoadjuvant induction chemotherapy followed by surgery, stratified by pathological complete response (pT0N0) and pT0‐1 tumor status.

Characteristic (*n*, %)	Induction chemotherapy plus surgery				Induction chemotherapy plus surgery			
	pCR (pT0N0)^a^ (*n* = 45) (*n*, %)	No pCR (*n* = 770) (*n*, %)	SMD (%)	*p*	pT0‐1 (*n* = 222) (*n*, %)	No pT0‐1 (*n* = 593) (*n*, %)	SMD (%)	*p*
Tumor subsite								
Tongue (215, 26.4)	14 (6.5)	201 (93.5)	11.10	0.2612	52 (24.2)	163 (75.8)	−9.34	0.1394
Buccal (308, 37.8)	20 (6.5)	288 (93.5)	14.36		96 (31.2)	212 (68.8)	15.37	
Other sites (292, 35.8)	11 (3.8)	281 (96.2)	−26.41		74 (25.3)	218 (74.7)	−7.19	
Sex								
Men (770, 94.5)	41 (5.3)	729 (94.7)	−13.91	0.3089	205 (26.6)	565 (73.4)	−12.21	0.1023
Women (45, 5.5)	4 (8.9)	41 (91.1)	13.91		17 (37.8)	28 (62.2)	12.21	
Age, years (mean ± SD)	54.29 ± 11.34	53.56 ± 9.88	6.86	0.6346	53.59 ± 10.78	53.61 ± 9.64	−0.20	0.9764
CCI (mean ± SD)	0.62 ± 0.91	0.57 ± 0.91	5.49	0.7095	0.57 ± 0.85	0.57 ± 0.93	0.00	0.9857
Clinical T status								
T1 (19, 2.3)	1 (5.3)	18 (94.7)	−0.77	0.1535	12 (63.2)	7 (36.8)	23.84	<0.0001
T2 (114, 14)	11 (9.6)	103 (90.4)	28.55		53 (46.5)	61 (53.5)	36.71	
T3 (83, 10.2)	7 (8.4)	76 (91.6)	17.13		28 (33.7)	55 (66.3)	10.71	
T4a (420, 51.5)	18 (4.3)	402 (95.7)	−24.68		92 (21.9)	328 (78.1)	−28.03	
T4b (179, 22)	8 (4.5)	171 (95.5)	−11.09		37 (20.7)	142 (79.3)	−18.17	
Clinical N status								
N0 (205, 25.2)	15 (7.3)	190 (92.7)	19.17	0.3797	67 (32.7)	138 (67.3)	15.66	0.0238
N1 (132, 16.2)	9 (6.8)	123 (93.2)	10.50		44 (33.3)	88 (66.7)	13.19	
N2 (441, 54.1)	20 (4.5)	421 (95.5)	−20.57		102 (23.1)	339 (76.9)	−22.60	
N3 (37, 4.5)	1 (2.7)	36 (97.3)	−13.47		9 (24.3)	28 (75.7)	−3.26	
Clinical stage								
I (13, 1.6)	1 (7.7)	12 (92.3)	4.88	0.1802	8 (61.5)	5 (38.5)	18.80	<0.0001
II (38, 4.7)	5 (13.2)	33 (86.8)	25.82		21 (55.3)	17 (44.7)	27.68	
III (61, 7.5)	4 (6.6)	57 (93.4)	5.44		24 (39.3)	37 (60.7)	16.42	
IV (703, 86.3)	35 (5.0)	668 (95.0)	−23.66		169 (24.0)	534 (76.0)	−37.80	
Use of a free flap								
No (213, 26.1)	24 (11.3)	189 (88.7)	61.79	<0.0001	92 (43.2)	121 (56.8)	46.74	<0.0001
Yes (602, 73.9)	21 (3.5)	581 (96.5)	−61.79		130 (21.6)	472 (78.4)	−46.74	
Adjuvant therapy after surgery								
None (169, 20.7)	24 (14.2)	145 (85.8)	76.98	<0.0001	79 (46.7)	90 (53.3)	48.24	<0.0001
Chemotherapy (46, 5.6)	7 (15.2)	39 (84.8)	35.02		25 (54.3)	21 (45.7)	29.81	
Radiotherapy (241, 29.6)	5 (2.1)	236 (97.9)	−49.52		50 (20.7)	191 (79.3)	−21.86	
Chemotherapy plus radiotherapy (359, 44)	9 (2.5)	350 (97.5)	−56.36		68 (18.9)	291 (81.1)	−38.35	

Abbreviations: CCI, Charlson comorbidity index; pCR, pathological complete response; SD, standard deviation; SMD, standardized mean difference.

^a^ pT0: the initial biopsy results confirmed the presence of squamous cell carcinoma; however, subsequent surgery revealed no residual tumor tissue.

### 2.7 Univariable and multivariable Cox regression analysis in the propensity score‐matched cohort

The results from both univariable and multivariable analyses conducted on the PS‐matched cohort are presented in Table 4. Both analyses indicated that undergoing IC + OP was not a significant risk factor for 5‐year DSS and OS.

**TABLE 4:** Univariable and multivariable analyses of risk factors for 5‐year disease‐specific survival and overall survival in the propensity score‐matched cohort.

Risk factor	Disease‐specific survival				Overall survival			
	Univariable analysis		Stepwise multivariable analysis		Univariable analysis		Stepwise multivariable analysis	
	HR (95% CI)	*p*	HR (95% CI)	*p*	HR (95% CI)	*p*	HR (95% CI)	*p*
Treatment								
Initial surgery	1		—		1		—	
Induction chemotherapy + surgery	1.11 (0.98–1.26)	0.1164	—	ns	1.00 (0.89–1.13)	1.0000	—	ns
Site								
Tongue	1.20 (1.05–1.36)	0.0058	1.26 (1.09–1.46)	0.0019	1.16 (1.03–1.30)	0.0129	1.26 (1.10–1.44)	0.0007
Buccal	0.87 (0.77–0.99)	0.0324	1.01 (0.88–1.16)	0.9385	0.86 (0.76–0.96)	0.0061	1.04 (0.92–1.18)	0.5031
Other sites	1		1		1		1	
Sex								
Male	0.99 (0.78–1.25)	0.9043	—	ns	1.01 (0.82–1.25)	0.9452	—	ns
Female	1		—		1		—	
Age, years	1.003 (0.998–1.008)	0.2888	1.008 (1.002–1.014)	0.0066	1.008 (1.003–1.013)	0.0009	1.012 (1.007–1.018)	<0.0001
CCI	1.04 (0.99–1.10)	0.1455	—	ns	1.10 (1.05–1.16)	<0.0001	1.10 (1.04–1.17)	0.0009
Clinical T status								
T1	1		1		1		1	
T2	1.69 (1.03–2.79)	0.0386	1.08 (0.59–2.00)	0.7969	1.95 (1.22–3.13)	0.0052	1.31 (0.75–2.30)	0.3445
T3	1.72 (1.04–2.87)	0.0362	1.05 (0.56–1.96)	0.8853	2.18 (1.35–3.51)	0.0014	1.31 (0.74–2.34)	0.3569
T4a	2.55 (1.58–4.12)	0.0001	1.46 (0.80–2.66)	0.2187	3.01 (1.91–4.73)	<0.0001	1.66 (0.95–2.89)	0.0766
T4b	2.63 (1.62–4.29)	0.0001	1.69 (0.92–3.10)	0.0934	3.09 (1.95–4.89)	<0.0001	1.68 (0.94–2.98)	0.0782
Clinical N status								
N0	1		—		1		—	
N1	1.38 (1.15–1.67)	0.0006	—	ns	1.39 (1.18–1.64)	<0.0001	—	ns
N2	1.82 (1.59–2.10)	<0.0001	—	ns	1.78 (1.57–2.01)	<0.0001	—	ns
N3	3.34 (2.63–4.26)	<0.0001	—	ns	3.57 (2.86–4.45)	<0.0001	—	ns
Clinical Stage								
I	1		—		1		—	
II	1.75 (0.82–3.77)	0.1498	—	ns	2.16 (1.06–4.38)	0.0341	—	ns
III	2.32 (1.12–4.81)	0.0233	—	ns	2.88 (1.46–5.69)	0.0024	—	ns
IV	4.05 (2.02–8.11)	<0.0001	—	ns	4.75 (2.47–9.15)	<0.0001	—	ns
Margin status								
Positive	2.10 (1.79–2.47)	<0.0001	1.64 (1.37–1.95)	<0.0001	2.05 (1.77–2.38)	<0.0001	1.57 (1.34–1.84)	<0.0001
<5 mm	1.31 (1.17–1.47)	<0.0001	1.24 (1.10–1.40)	0.0005	1.25 (1.12–1.39)	<0.0001	1.20 (1.08–1.34)	0.0010
≥5 mm	1		1		1		1	
Differentiation								
Well differentiated	1				1		1	
Moderated differentiated	1.69 (1.47–1.95)	<0.0001	1.31 (1.12–1.53)	0.0006	1.55 (1.37–1.75)	<0.0001	1.20 (1.05–1.38)	0.0072
Poorly differentiated	2.96 (2.45–3.57)	<0.0001	1.89 (1.54–2.32)	<0.0001	2.64 (2.22–3.12)	<0.0001	1.78 (1.48–2.14)	<0.0001
Pathologic T status								
T0	1				1			
T1	0.94 (0.59–1.51)	0.8096	—		0.85 (0.56–1.29)	0.4514	1	
T2	1.30 (0.84–2.02)	0.2359	—	ns	1.25 (0.85–1.83)	0.2601	1.19 (0.92–1.55)	0.1849
T3	1.62 (1.04–2.53)	0.0328	—	ns	1.61 (1.09–2.37)	0.0165	1.43 (1.09–1.86)	0.0097
T4a	1.99 (1.30–3.04)	0.0015	—	ns	1.94 (1.34–2.81)	0.0004	1.74 (1.37–2.21)	<0.0001
T4b	2.32 (1.50–3.61)	0.0002	—	ns	2.38 (1.62–3.50)	<0.0001	2.08 (1.58–2.72)	<0.0001
Pathologic N status								
pNx	1				1		1	
pN0	0.78 (0.55–1.10)	0.1597	1.004 (0.52–1.96)	0.9901	0.75 (0.56–1.01)	0.0608	1.10 (0.64–1.89)	0.7295
pN1	1.76 (1.21–2.54)	0.0028	2.16 (1.08–4.30)	0.0290	1.50 (1.10–2.06)	0.0115	2.17 (1.24–3.79)	0.0066
pN2	2.87 (2.04–4.06)	<0.0001	3.02 (1.53–5.97)	0.0014	2.58 (1.93–3.45)	<0.0001	3.69 (2.14–6.36)	<0.0001
pN3	4.19 (2.90–6.05)	<0.0001	3.84 (1.92–7.69)	0.0001	3.81 (2.77–5.23)	<0.0001	4.56 (2.60–8.00)	<0.0001
Pathologic stage								
I	1		1		1		—	
II	1.32 (0.88–1.98)	0.1870	1.21 (0.75–1.95)	0.4341	1.44 (1.01–2.04)	0.0430	—	ns
III	1.92 (1.31–2.83)	0.0009	1.27 (0.79–2.05)	0.3320	1.98 (1.41–2.77)	<0.0001	—	ns
IV	4.13 (2.93–5.80)	<0.0001	1.79 (1.14–2.80)	0.0108	4.12 (3.06–5.55)	<0.0001	—	ns
Adjuvant therapy after surgery								
None	1		1		1		1	
Chemotherapy	1.29 (0.96–1.73)	0.0913	0.84 (0.60–1.18)	0.3169	1.29 (0.995–1.67)	0.0549	0.82 (0.61–1.10)	0.1879
Radiotherapy	1.12 (0.94–1.34)	0.1924	0.82 (0.67–0.998)	0.0475	1.08 (0.92–1.26)	0.3525	0.76 (0.64–0.91)	0.0022
Chemotherapy plus radiotherapy	1.63 (1.42–1.87)	<0.0001	0.71 (0.60–0.83)	<0.0001	1.56 (1.38–1.77)	<0.0001	0.68 (0.59–0.79)	<0.0001

Abbreviations: CCI, Charlson comorbidity index; CI, confidence interval; HR, hazard ratio; ns, not significant.

## 3 DISCUSSION

In this PS‐matched analysis, the IC + OP group included a higher proportion of patients with pT0–1 tumors and p‐stage I disease. Furthermore, 28.4% ([721–516]/721) of patients with stage IV disease experienced downstaging after IC. Although this did not confer a survival benefit, it did stratify patients for further adjuvant treatment. This indicates that IC predominantly impacts the primary tumor, rather than the neck nodal disease. This is also reflected in the fact that fewer patients in the IC + OP group required free flap reconstruction and fewer received postoperative CRT. While the 5‐year outcomes for the OP group and the IC + OP group were comparable, we found that in pT4a tumors, the OP group had better 5‐year outcomes than the IC + OP group, with DSS at 62% versus 52%, and OS at 53% versus 44%, respectively.

IC has the potential to downstage unresectable bulky tumors to a resectable status, control tumor progression prior to surgery, preserve organs, and reduce the risk of distant metastases. Furthermore, the pathological response to chemotherapy can stratify further adjuvant treatment in high‐risk patients prone to treatment failure.^17^ Previous studies have reported resectability rates ranging from 19% to 43% for OCSCC following IC.^3^, ^4^, ^5^, ^6^ Notably, IC plays a crucial role in reducing surgical morbidities, primarily by enabling preservation of oral organs through conservative surgery. Lee et al.^7^ have previously shown that 70% (16/23) of patients with tongue SCC could undergo tongue conservation treatment following IC. Similarly, Abdelmeguid et al.^8^ reported that 25% (15/60) of the OCSCC surgeries were less extensive than initially planned. Although the current study could not assess detailed data on conservative surgery rates, the PS‐matched group revealed that fewer patients in the IC + OP group required free flap reconstruction. Consequently, it is reasonable to hypothesize that more conservative procedures would reduce the need for free flap reconstruction. In studies focusing on IC, groups with a pCR have consistently shown more favorable outcomes compared to other groups.^7^, ^11^ The present study demonstrated that, in the PS‐matched cohort, patients who achieved pCR exhibited superior DSS (95% vs. 60%) and OS (95% vs. 54%). However, due to the inherent limitations of this nationwide study—which are also applicable to the Surveillance, Epidemiology, and End Results (SEER) and National Cancer Database (NCDB)—DFS cannot be accurately represented.^18^, ^19^ Although pCR rates in small case studies of neoadjuvant IC appear high, ranging from 27% to 50%, the current investigation found a pCR rate of only 6%. This discrepancy may be due to the fact that not all patients received neoadjuvant chemotherapy. However, a notable constraint of this study is the inability to distinguish between patients who received IC as a bridging therapy or as a neoadjuvant therapy, due to the TCRD's lack of data on specific chemotherapy regimens. As a result, we are unable to draw definitive conclusions on whether outcomes differed for patients who received systemic therapy as neoadjuvant or as bridge therapy. However, we have mitigated this limitation by excluding patients who received IC within the 30 days preceding surgery. It is plausible that this group of patients received oral chemotherapy with minimal influence on survival outcomes. Surprisingly, in this real‐world practice study, more than 700 patients received IC within 30 days before surgery (Figure 1).

In our study, a mere 1.6% (13/833) of patients in the IC + OP group had stage I disease. In contrast, 28.5% (8280/29058) of patients in the OP group had stage I disease. The precise reason for administering induction chemotherapy in patients with stage I tumors was not specified in the database. Potential explanations include initial patient refusal of surgery or a delayed surgical schedule. Notably, 23.6% (197/833) of patients in the IC + OP group had T4b tumors, whereas only 3.3% (965/29058) in the OP group harbored T4b malignancies. Despite the smaller proportion of T4b tumors in the OP group, it still had a larger number of surgical patients (*n* = 965) compared to the IC + OP group. It is important to highlight that the current treatment modalities for upfront surgery in T4b tumors remain controversial. The AJCC sixth edition, published in 2002, defined T4b OCSCC as an unresectable tumor. However, the AJCC seventh edition, released in 2010, modified the definition of T4b OCSCC to very advanced local disease. The most recent NCCN guidelines, updated in 2024, continue to recommend clinical trials or nonsurgical approaches for the treatment of T4b OCSCC tumors.^1^ However, our previous study, published in 2007, demonstrated that resected T4b tumors located below the mandibular notch (infra‐notch T4b, accounting for 85% of T4b tumors) had better survival outcomes compared to tumors above the notch (supra‐notch T4b, accounting for 15% of T4b tumors). Specifically, the 5‐year DFS rates were 65% for infra‐notch T4b tumors and 14% for supra‐notch T4b tumors (*p* = 0.0004).^20^ Based on these findings, we have since adopted a practice of performing routine upfront surgery for infra‐notch T4b tumors in our patients. This anatomical guideline (infra‐notch vs. supra‐notch) has now been widely adopted by several studies to determine the resectability of T4b tumors.^21^, ^22^, ^23^ In another investigation we previously conducted on Taiwanese patients with T4b tumors, we found that primary surgery was performed in 66% (327/492) of clinical T4b (cT4b) OCSCC, with clinical N0‐2 (cN0‐2) cases showing a favorable prognosis.^24^ It is important to recognize that the decision to perform upfront surgery for T4b tumors is not only guided by the anatomical location but also depends on the resection and reconstruction capabilities of each head and neck surgical team.

A meta‐analysis of 27 randomized trials involving 2872 patients revealed that IC does not significantly improve OS, DSS, or distant metastasis rates. However, it did show a significant improvement in locoregional recurrence.^25^ Another meta‐analysis, which compared two phase III randomized studies of IC followed by surgery with or without postoperative RT to surgery with or without postoperative RT, found no significant overall benefit in favor of IC regarding locoregional recurrence, disease‐free survival, and OS rates. Nevertheless, a subgroup analysis of individual data from cN2 patients demonstrated a statistically significant OS benefit in favor of IC.^26^ In the current study, we were unable to present an analysis of locoregional recurrence due to the inherent limitations of the TCRD. While no significant differences in terms of survival were observed in subgroups with cT4a tumors, cT4b tumors, cN3 disease, pT4b tumors, and pN3 disease, the OP group exhibited superior 5‐year outcomes in the case of pT4a tumors. Several factors may explain the inferior outcomes observed in patients with pT4a tumors in the IC + OP group. These include well‐differentiated tumors, the presence of lesions in the buccal subsite, male sex, and absence of nodal involvement. In the context of head and neck cancer, well‐differentiated squamous cell carcinoma has been found to be relatively less sensitive to IC than poorly differentiated tumors.^27^ Recent advancements in next‐generation sequencing (NGS) have revealed that mutations in the *FAT1* gene and the subsequent activation of the Wnt/β‐catenin signaling pathway may contribute to chemoresistance in well‐differentiated squamous cell carcinoma compared to poorly differentiated tumors.^28^ With regard to tumor subsite, buccal cancer is known to be a highly aggressive form of OCSCC, often presenting with bulky volumes and a high rate of local recurrence.^29^, ^30^ In general, buccal cancer is more prevalent in men and accounts for 4%–7% of all head and neck squamous cell carcinomas in the United States. However, its prevalence is markedly higher in India and Taiwan, reaching up to 37%.^31^, ^32^, ^33^, ^34^ While tobacco and alcohol consumption are the primary risk factors for buccal squamous cell carcinoma in the United States, the practice of betel quid chewing in Southeast Asia significantly contributes to the higher incidence of this malignancy. This is attributed to the chronic exposure of the buccal mucosa to carcinogens and the persistent local inflammation induced by betel quid chewing.^35^ This oral habit, which is more prevalent among men, may also explain the sex disparity in the prevalence of OCSCC at this specific subsite. Notably, NGS studies conducted in OCSCC patients from betel quid chewing endemic areas have revealed unique genetic mutations, affecting the *USP9X*, *MLL4*, *ARID2*, *UNC13C*, and TRPM3 genes, beyond the common pathogenic variants in the *TP53*, *FAT1*, *CASP8*, *HRAS*, and *NOTCH1* genes.^36^ Interestingly, a specific mutation‐based signature in OCSCC has been shown to predict outcomes in a Taiwanese study.^37^ These findings may help elucidate the mechanisms behind the observed chemoresistance and poorer outcomes in the IC + OP group compared to the OP group in patients with pT4a tumors. This suggests that more aggressive adjuvant therapy may be needed after surgery for this group of patients. The recent advent of innovative therapeutic strategies, such as targeted therapies and immune checkpoint inhibitors, has shown promising anti‐cancer effects in the treatment of recurrent or metastatic head and neck squamous cell carcinomas.^38^ When these approaches are combined with IC or applied after surgery, they hold the potential to amplify treatment responses in patients with locally advanced OCSCC. If these strategies prove successful in clinical trials, they could lay the groundwork for more effective clinical management in the future. Although we attempted to identify subpopulations likely to respond to IC, our analysis revealed that only patients with cT2–3, cN1, and c‐Stage II disease were more likely to achieve pT0–1 status. Notably, no subpopulation of OP + IC patients was found to significantly achieve pCR. It is important to acknowledge that we were unable to assess the potential confounding effect of human papillomavirus (HPV) infection status on our findings. Although the role of HPV infections in the pathogenesis of OCSCC is not yet fully understood, our previous research has demonstrated that HPV 16/18 E7 viral loads can predict the risk of distant metastases in patients with OCSCC.^39^, ^40^, ^41^ Further *investigation* is warranted to elucidate the potential relationship between HPV infections and the timing of tumor relapse.

This study has several limitations that should be considered. Firstly, being a registry‐based nationwide investigation, it is prone to unmeasured confounding factors. These unaccounted factors could introduce bias, potentially affecting the validity of our results. Secondly, the study was conducted in a region with a high prevalence of betel nut chewing, a cultural practice that significantly contributes to a high occurrence of buccal SCC. This factor may limit the applicability of our findings to Western countries. Lastly, the absence of detailed information on specific chemotherapy regimens implies that the IC + OP group might include patients who received neoadjuvant and bridging therapy. It is important to note that induction chemotherapy and bridging chemotherapy are distinct forms of treatment. Unfortunately, due to the limitations of the available data, these two groups had to be combined in the current study, potentially obscuring any differences in outcomes between them. Although cisplatin‐based chemotherapy regimens are a cornerstone of systemic treatment for head and neck cancer,^1^ the lack of detailed information on the specific schemes employed in our study precluded an assessment of whether all patients received the same chemotherapy protocol. Consequently, we are unable to determine if variations in protocols might have influenced outcomes.

## 4 CONCLUSIONS

In summary, this study, based on PS matching, showed that the IC + OP group benefits primarily from primary tumor regression rather than nodal regression. This group also had fewer patients requiring postoperative CRT and free flap reconstruction. While the survival rates of the IC + OP group were comparable to the OP group, this was not the case for the subgroup with pT4a tumors. These findings highlight the nuanced role of IC in managing OCSCC, suggesting its benefits may be tumor‐stage specific. Future clinical trials could help enhance treatment responses in pT4a OCSCC. While IC may not universally improve survival, it could be advantageous for patients who respond positively to the treatment.

## AUTHOR CONTRIBUTIONS

**Cheng‐Lung Hsu:** Conceptualization (lead); data curation (lead); formal analysis (lead); investigation (lead); methodology (lead); project administration (lead); resources (lead); software (lead); supervision (lead); validation (lead); visualization (lead); writing – original draft (lead); writing – review and editing (equal). **Yu‐Wen Wen:** Conceptualization (equal); data curation (equal); formal analysis (equal); funding acquisition (lead); investigation (equal); methodology (equal); project administration (equal); resources (equal); software (equal); supervision (equal); validation (equal); visualization (equal); writing – original draft (equal). **Hung‐Ming Wang:** Conceptualization (equal); data curation (equal); formal analysis (equal); investigation (equal); methodology (equal); project administration (equal); resources (equal); software (equal); supervision (equal); validation (equal); visualization (equal); writing – original draft (equal). **Chia‐Hsun Hsieh:** Conceptualization (equal); data curation (equal); formal analysis (equal); investigation (equal); methodology (equal); project administration (equal); resources (equal); software (equal); supervision (equal); validation (equal); visualization (equal); writing – original draft (equal). **Chi‐Ting Liao:** Conceptualization (equal); data curation (equal); formal analysis (equal); investigation (equal); methodology (equal); project administration (equal); resources (equal); software (equal); supervision (equal); validation (equal); visualization (equal); writing – original draft (equal). **Li‐Yu Lee:** Conceptualization (equal); data curation (equal); formal analysis (equal); investigation (equal); methodology (equal); project administration (equal); resources (equal); software (equal); supervision (equal); validation (equal); visualization (equal); writing – original draft (equal). **Shu‐Hang Ng:** Conceptualization (equal); data curation (equal); formal analysis (equal); investigation (equal); methodology (equal); project administration (equal); resources (equal); software (equal); supervision (equal); validation (equal); visualization (equal); writing – original draft (equal). **Chien‐Yu Lin:** Conceptualization (equal); data curation (equal); formal analysis (equal); investigation (equal); methodology (equal); project administration (equal); resources (equal); software (equal); supervision (equal); validation (equal); visualization (equal); writing – original draft (equal). **Wen‐Cheng Chen:** Conceptualization (equal); data curation (equal); formal analysis (equal); investigation (equal); methodology (equal); project administration (equal); resources (equal); software (equal); supervision (equal); validation (equal); visualization (equal); writing – original draft (equal). **Jin‐Ching Lin:** Conceptualization (equal); data curation (equal); formal analysis (equal); investigation (equal); methodology (equal); project administration (equal); resources (equal); software (equal); supervision (equal); validation (equal); visualization (equal); writing – original draft (equal). **Yao‐Te Tsai:** Conceptualization (equal); data curation (equal); formal analysis (equal); investigation (equal); methodology (equal); project administration (equal); resources (equal); software (equal); supervision (equal); validation (equal); visualization (equal); writing – original draft (equal). **Shu‐Ru Lee:** Conceptualization (equal); data curation (equal); formal analysis (equal); investigation (equal); methodology (equal); project administration (equal); resources (equal); software (equal); supervision (equal); validation (equal); visualization (equal); writing – original draft (equal). **Chih‐Yen Chien:** Conceptualization (equal); data curation (equal); formal analysis (equal); investigation (equal); methodology (equal); project administration (equal); resources (equal); software (equal); supervision (equal); validation (equal); visualization (equal); writing – original draft (equal). **Chun‐Hung Hua:** Conceptualization (equal); data curation (equal); formal analysis (equal); investigation (equal); methodology (equal); project administration (equal); resources (equal); software (equal); supervision (equal); validation (equal); visualization (equal); writing – original draft (equal). **Cheng Ping Wang:** Conceptualization (equal); data curation (equal); formal analysis (equal); investigation (equal); methodology (equal); project administration (equal); resources (equal); software (equal); supervision (equal); validation (equal); visualization (equal); writing – original draft (equal). **Tsung‐Ming Chen:** Conceptualization (equal); data curation (equal); formal analysis (equal); investigation (equal); methodology (equal); project administration (equal); resources (equal); software (equal); supervision (equal); validation (equal); visualization (equal); writing – original draft (equal). **Shyuang‐Der Terng:** Conceptualization (equal); data curation (equal); formal analysis (equal); investigation (equal); methodology (equal); project administration (equal); resources (equal); software (equal); supervision (equal); validation (equal); visualization (equal); writing – original draft (equal). **Chi‐Ying Tsai:** Conceptualization (equal); data curation (equal); formal analysis (equal); investigation (equal); methodology (equal); project administration (equal); resources (equal); software (equal); supervision (equal); validation (equal); visualization (equal); writing – original draft (equal). **Kang‐Hsing Fan:** Conceptualization (equal); data curation (equal); formal analysis (equal); investigation (equal); methodology (equal); project administration (equal); resources (equal); software (equal); supervision (equal); validation (equal); visualization (equal); writing – original draft (equal). **Chih‐Hua Yeh:** Conceptualization (equal); data curation (equal); formal analysis (equal); investigation (equal); methodology (equal); project administration (equal); resources (equal); software (equal); supervision (equal); validation (equal); visualization (equal); writing – original draft (equal). **Chih‐Hung Lin:** Conceptualization (equal); data curation (equal); formal analysis (equal); investigation (equal); methodology (equal); project administration (equal); resources (equal); software (equal); supervision (equal); validation (equal); visualization (equal); writing – original draft (equal). **Chung‐Kan Tsao:** Conceptualization (equal); data curation (equal); formal analysis (equal); investigation (equal); methodology (equal); project administration (equal); resources (equal); software (equal); supervision (equal); validation (equal); visualization (equal); writing – original draft (equal). **Nai‐Ming Cheng:** Conceptualization (equal); data curation (equal); formal analysis (equal); investigation (equal); methodology (equal); project administration (equal); resources (equal); software (equal); supervision (equal); validation (equal); visualization (equal); writing – original draft (equal). **Tuan‐Jen Fang:** Conceptualization (equal); data curation (equal); formal analysis (equal); investigation (equal); methodology (equal); project administration (equal); resources (equal); software (equal); supervision (equal); validation (equal); visualization (equal); writing – original draft (equal). **Shiang‐Fu Huang:** Conceptualization (equal); data curation (equal); formal analysis (equal); investigation (equal); methodology (equal); project administration (equal); resources (equal); software (equal); supervision (equal); validation (equal); visualization (equal); writing – original draft (equal). **Chung‐Jan Kang:** Conceptualization (equal); data curation (equal); formal analysis (equal); investigation (equal); methodology (equal); project administration (equal); resources (equal); software (equal); supervision (equal); validation (equal); visualization (equal); writing – original draft (equal). **Li‐Ang Lee:** Conceptualization (equal); data curation (equal); formal analysis (equal); investigation (equal); methodology (equal); project administration (equal); resources (equal); software (equal); supervision (equal); validation (equal); visualization (equal); writing – original draft (equal). **Ku‐Hao Fang:** Conceptualization (equal); data curation (equal); formal analysis (equal); investigation (equal); methodology (equal); project administration (equal); resources (equal); software (equal); supervision (equal); validation (equal); visualization (equal); writing – original draft (equal). **Yu‐Chien Wang:** Conceptualization (equal); data curation (equal); formal analysis (equal); investigation (equal); methodology (equal); project administration (equal); resources (equal); software (equal); supervision (equal); validation (equal); visualization (equal); writing – original draft (equal). **Wan‐Ni Lin:** Conceptualization (equal); data curation (equal); formal analysis (equal); investigation (equal); methodology (equal); project administration (equal); resources (equal); software (equal); supervision (equal); validation (equal); visualization (equal); writing – original draft (equal). **Li‐Jen Hsin:** Conceptualization (equal); data curation (equal); formal analysis (equal); investigation (equal); methodology (equal); project administration (equal); resources (equal); software (equal); supervision (equal); validation (equal); visualization (equal); writing – original draft (equal). **Tzu‐Chen Yen:** Conceptualization (equal); data curation (equal); formal analysis (equal); investigation (equal); methodology (equal); project administration (equal); resources (equal); software (equal); supervision (equal); validation (equal); visualization (equal); writing – original draft (equal). **Chun‐Ta Liao:** Conceptualization (lead); data curation (lead); formal analysis (lead); investigation (lead); methodology (lead); project administration (lead); resources (lead); software (lead); supervision (lead); validation (lead); visualization (lead); writing – original draft (lead); writing – review and editing (lead).

## FUNDING INFORMATION

This research received financial support through grants (CMRPD1H0521 and BMRPC55) provided by the Chang Gung Medical Research Program.

## CONFLICT OF INTEREST STATEMENT

The authors declare that there are no conflicts of interest that could potentially influence the presentation or interpretation of the research findings in this study.

## Supporting information


Figure S1.

## Data Availability

The availability of data used in this study is subject to third‐party restrictions imposed by the Health and Welfare Data Center of the Taiwanese Ministry of Health and Welfare (http: //dep. mohw.gov.tw/DOS/), in accordance with the “Personal Information Protection Act.” Despite these limitations, the authors were granted a license to utilize the data for the purposes of this research. Access to the datasets generated and analyzed during this study can be provided by the corresponding author upon reasonable request. However, this is contingent on obtaining formal permission from the Taiwanese Ministry of Health and Welfare.

## References

[1] National Comprehensive Cancer Network Clinical Practice Guidelines in Oncology, Head and Neck Cancers, Version 4.2024. Accessed May 20, 2024. http://www.nccn.org/

[2] Lin CY , Fan KH , Lee LY , et al. Precision adjuvant therapy based on detailed pathological risk factors for resected oral cavity squamous cell carcinoma: long term outcome comparison of CGMH and NCCN guidelines. Int J Radiat Oncol Biol Phys. 2020;106(5):916‐925. doi:10.1016/j.ijrobp.2019.08.058 31499138

[3] Rudresha AH , Chaudhuri T , Lakshmaiah KC , et al. Induction chemotherapy in TechnicallyUnresectable locally advanced T4a Oral cavity squamous cell cancers: experience from a regional cancer Center of South India. Indian J Med Paediatr Oncol. 2017;38(4):490‐494. doi:10.4103/ijmpo.ijmpo_185_16 29333018 PMC5759070

[4] Rudresha AH , Chaudhuri T , Lakshmaiah KC , et al. Induction chemotherapy in locally advanced T4b oral cavity squamous cell cancers: a regional cancer center experience. Indian J Cancer. 2017;54(1):35‐38. doi:10.4103/ijc.IJC_131_17 29199659

[5] Patil VM , Prabhash K , Noronha V , et al. Neoadjuvant chemotherapy followed by surgery in very locally advanced technically unresectable oral cavity cancers. Oral Oncol. 2014;50(10):1000‐1004. doi:10.1016/j.oraloncology.2014.07.015 25130412

[6] Joshi A , Patil VM , Noronha V , et al. Is there a role of induction chemotherapy followed by resection in T4b oral cavity cancers? Indian J Cancer. 2013;50(4):349‐355. doi:10.4103/0019-509X.123627 24369216

[7] Lee TL , Wei PY , Yang MH , Chang PM , Wang LW , Tai SK . Tongue conservation treatment for oral tongue squamous cell carcinoma with induction chemotherapy, surgery, and risk‐adapted adjuvant therapy: a phase II trial. Cancer Rep (Hoboken). 2022;5(2):e1456. doi:10.1002/cnr2.1456 34051137 PMC8842695

[8] Abdelmeguid AS , Silver NL , Boonsripitayanon M , et al. Role of induction chemotherapy for oral cavity squamous cell carcinoma. Cancer. 2021;127(17):3107‐3112. doi:10.1002/cncr.33616 33909292

[9] Geiger JL , Adelstein DJ . Chemotherapy in the definitive management of oral cancers: where do we stand today? Oral Oncol. 2020;102:104584. doi:10.1016/j.oraloncology.2020.104584 32032863

[10] Licitra L , Grandi C , Guzzo M , et al. Primary chemotherapy in resectable oral cavity squamous cell cancer: a randomized controlled trial. J Clin Oncol. 2003;21(2):327‐333. doi:10.1200/JCO.2003.06.146 12525526

[11] Bossi P , Lo Vullo S , Guzzo M , et al. Preoperative chemotherapy in advanced resectable OCSCC: long‐term results of a randomized phase III trial. Ann Oncol. 2014;25(2):462‐466. doi:10.1093/annonc/mdt555 24401930

[12] Fu JY , Yue XH , Dong MJ , Li J , Zhang CP . Assessment of neoadjuvant chemotherapy with docetaxel, cisplatin, and fluorouracil in patients with oral cavity cancer. Cancer Med. 2023;12(3):2417‐2426. doi:10.1002/cam4.5075 35880556 PMC9939210

[13] McShane LM , Altman DG , Sauerbrei W , Taube SE , Gion M , Clark GM . REporting recommendations for tumour MARKer prognostic studies (REMARK). Br J Cancer. 2005;93(4):387‐391. doi:10.1038/sj.bjc.6602678 16106245 PMC2361579

[14] Sauerbrei W , Taube SE , McShane LM , Cavenagh MM , Altman DG . Reporting recommendations for tumor marker prognostic studies (REMARK): an abridged explanation and elaboration. J Natl Cancer Inst. 2018;110(8):803‐811. doi:10.1093/jnci/djy088 29873743 PMC6093349

[15] Chien CY , Wang CP , Lee LY , et al. Indications for elective neck dissection in cT1N0M0 oral cavity cancer according to the AJCC eight edition: a nationwide study. Oral Oncol. 2023;140:106366. doi:10.1016/j.oraloncology.2023.106366 36965411

[16] American College of Surgeons. Standards for Oncology Registry Entry. STORE 2024. Effective for cases diagnosed. 2024 Accessed May 20, 2024. https://wwwfacsorg/media/vssjur3j/store_manual_2024pdf

[17] Marta GN , William WN Jr , Feher O , Carvalho AL , Kowalski LP . Induction chemotherapy for oral cavity cancer patients: current status and future perspectives. Oral Oncol. 2015;51(12):1069‐1075. doi:10.1016/j.oraloncology.2015.10.009 26522693

[18] Tsai CY , Wen YW , Lee SR , et al. Early relapse is an adverse prognostic factor for survival outcomes in patients with oral cavity squamous cell carcinoma: results from a nationwide registry study. BMC Cancer. 2023;23(1):126. doi:10.1186/s12885-023-10602-1 36750965 PMC9906940

[19] Janz TA , Graboyes EM , Nguyen SA , et al. A comparison of the NCDB and SEER database for research involving head and neck cancer. Otolaryngol Head Neck Surg. 2019;160(2):284‐294. doi:10.1177/0194599818792205 30129822

[20] Liao CT , Ng SH , Chang JT , et al. T4b oral cavity cancer below the mandibular notch is resectable with a favorable outcome. Oral Oncol. 2007;43:570‐579. doi:10.1016/j.oraloncology.2006.06.008 16996777

[21] Trivedi NP . Oral cancer involving masticator space (T4b): review of literature and future directions. Head Neck. 2018;40(10):2288‐2294. doi:10.1002/hed.25211 29756367

[22] Asarkar AA , Chang BA , de Bree R , et al. Primary management of operable locally advanced oral cavity squamous cell carcinoma: current concepts and strategies. Adv Ther. 2024;41(6):2133‐2150. doi:10.1007/s12325-024-02861-6 38642199

[23] Mattavelli D , Montenegro C , Piazza C . Compartmental surgery for T4b oral squamous cell carcinoma involving the masticatory space. Curr Opin Otolaryngol Head Neck Surg. 2024;32(2):55‐61. doi:10.1097/MOO.0000000000000958 38193497 PMC10919272

[24] Liao CT , Wen YW , Lee SR , et al. Clinical outcomes of Taiwanese patients with cT4 oral cavity squamous cell crcinoma: toward the identification of the optimal initial treatment approach for cT4b patients. Ann Surg Oncol. 2017;24:785‐793. 27896513 10.1245/s10434-016-5629-x

[25] Lau A , Li KY , Yang WF , Su YX . Induction chemotherapy for squamous cell carcinomas of the oral cavity: a cumulative meta‐analysis. Oral Oncol. 2016;61:104‐114. doi:10.1016/j.oraloncology.2016.08.022 27688112

[26] Marta GN , Riera R , Bossi P , et al. Induction chemotherapy prior to surgery with or without postoperative radiotherapy for oral cavity cancer patients: systematic review and meta‐analysis. Eur J Cancer. 2015;51(17):2596‐2603. doi:10.1016/j.ejca.2015.08.007 26318725

[27] Nakashima T , Maehara Y , Kohnoe S , Hayashi I , Katsuta Y . Histologic differentiation and chemosensitivity of human head and neck squamous cell carcinomas. Head Neck. 1990;12(5):406‐410. doi:10.1002/hed.2880120506 2211101

[28] Kina S , Kawabata‐Iwakawa R , Miyamoto S , Arasaki A , Sunakawa H , Kinjo T . A molecular signature of well‐differentiated oral squamous cell carcinoma reveals a resistance mechanism to metronomic chemotherapy and novel therapeutic candidates. J Drug Target. 2021;29(10):1118‐1127. doi:10.1080/1061186X.2021.1929256 33979258

[29] Liao CT , Lee LY , Hsueh C , et al. Comparative outcomes in oral cavity cancer with resected pT4a and pT4b. Oral Oncol. 2013;49(3):230‐236. doi:10.1016/j.oraloncology.2012.09.010 23063612

[30] Diaz EM Jr , Holsinger FC , Zuniga ER , Roberts DB , Sorensen DM . Squamous cell carcinoma of the buccal mucosa: one institution's experience with 119 previously untreated patients. Head Neck. 2003;25(4):267‐273. doi:10.1002/hed.10221 12658730

[31] Stepan KO , Mazul AL , Larson J , et al. Changing epidemiology of Oral cavity cancer in the United States. Otolaryngol Head Neck Surg. 2023;168(4):761‐768. doi:10.1177/01945998221098011 35503657 PMC10154079

[32] Siegel R , Ma J , Zou Z , Jemal A . Cancer statistics, 2014. CA Cancer J Clin. 2014;64(1):9‐29. doi:10.3322/caac.21208 24399786

[33] Chen PC , Kuo C , Pan CC , Chou MY . Risk of oral cancer associated with human papillomavirus infection, betel quid chewing, and cigarette smoking in Taiwan—an integrated molecular and epidemiological study of 58 cases. J Oral Pathol Med. 2002;31(6):317‐322. doi:10.1034/j.1600-0714.2002.00129.x 12190813

[34] Liao CT , Wang HM , Ng SH , et al. Good tumor control and survivals of squamous cell carcinoma of buccal mucosa treated with radical surgery with or without neck dissection in Taiwan. Oral Oncol. 2006;42(8):800‐809. doi:10.1016/j.oraloncology.2005.11.020 16458038

[35] Chen YJ , Chang JT , Liao CT , et al. Head and neck cancer in the betel quid chewing area: recent advances in molecular carcinogenesis. Cancer Sci. 2008;99(8):1507‐1514. doi:10.1111/j.1349-7006.2008.00863.x 18754860 PMC11159516

[36] India project team of the international cancer genome consortium. Mutational landscape of gingivo‐buccal oral squamous cell carcinoma reveals new recurrently‐mutated genes and molecular subgroups. Nat Commun. 2013;4:2873. doi:10.1038/ncomms3873 24292195 PMC3863896

[37] Chen SJ , Liu H , Liao CT , et al. Ultra‐deep targeted sequencing of advanced oral squamous cell carcinoma identifies a mutation‐based prognostic gene signature. Oncotarget. 2015;6(20):18066‐18080. doi:10.18632/oncotarget.3768 25980437 PMC4621868

[38] Goel B , Tiwari AK , Pandey RK , et al. Therapeutic approaches for the treatment of head and neck squamous cell carcinoma‐an update on clinical trials. Transl Oncol. 2022;21:101426. doi:10.1016/j.tranon.2022.101426 35460943 PMC9046875

[39] Huang CG , Lee LA , Tsao KC , et al. Human papillomavirus 16/18 E7 viral loads predict distant metastasis in oral cavity squamous cell carcinoma. J Clin Virol. 2014;61:230‐236. doi:10.1016/j.jcv.2014.07.007 25097016

[40] Lee LA , Huang CG , Tsao KC , et al. Increasing rates of low‐risk human papillomavirus infections in patients with oral cavity squamous cell carcinoma: association with clinical outcomes. J Clin Virol. 2013;57:331‐337. doi:10.1016/j.jcv.2013.04.010 23669598

[41] Lee LA , Huang CG , Liao CT , et al. Human papillomavirus‐16 infection in advanced oral cavity cancer patients is related to an increased risk of distant metastases and poor survival. PLoS One. 2012;7:e40767. doi:10.1371/journal.pone.0040767 22808258 PMC3395633

