# Supplementary figures and images for: Prognostic impact of bridge or neoadjuvant induction chemotherapy in patients with resected oral cavity cancer: A nationwide cohort study
Source: Cancer Med. 2024 Aug 5;13(15):e70061. doi: 10.1002/cam4.70061 (PMC11299076; doi:10.1002/cam4.70061)

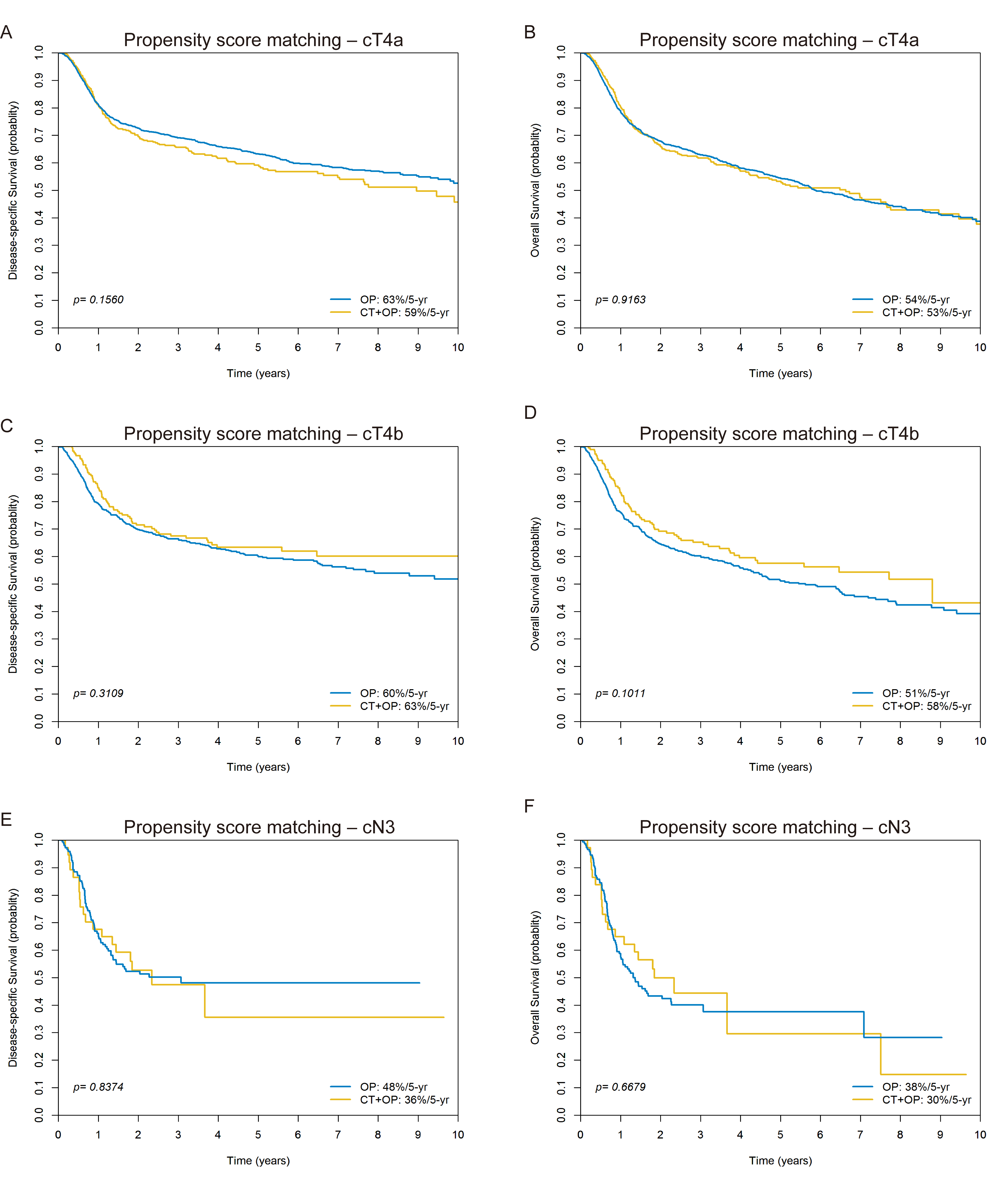

Supplement: Supplementary file 1 — Figure S1. [file CAM4-13-e70061-s001.tif]
